# Folding of the nonacylated acylation domain of the adenylate cyclase toxin is templated by its C-terminal RTX domain

**DOI:** 10.1016/j.jbc.2026.113451

**Published:** 2026-08-14

**Authors:** Guojun Chen, Carlos Espinosa-Vinals, Hongbin Li

**Affiliations:** 1 Department of Chemistry, University of British Columbia, Vancouver, Canada; 2 Institute of Microbiology of the Czech Academy of Sciences, Prague, Czech Republic

**Keywords:** Acylation domain, Adeynate Cyclase, Folding, Optical tweezers, Protein folding, Templated

## Abstract

Adenylate cyclase toxin (CyaA) is a key virulence factor secreted by the whooping cough-causing bacterium *Bordetella pertussis.* Its acylation domain (AD) is a conserved functional domain amongst various pore-forming toxins. In CyaA, AD connects the C-terminal repeats-in-toxin (RTX) domain to the N-terminal functional domains, such as the hydrophobic domain and the adenylate cyclase domain, and its proper folding is important for the acquisition of the active conformation and full toxin activity of CyaA. However, the mechanism of AD folding remains elusive. Using single-molecule optical tweezers, here we investigated the folding pathway of nonacylated AD in the context of a truncated RTX scaffold. We showed that a truncated RTX construct, ΔRTX-i/v, in which the N-terminal part of RTX-i was connected to the C-terminal part of RTX-v, is folded upon binding of Ca^2+^ and can scaffold the folding of the AD. In the absence of Ca^2+^, the AD was unfolded. Upon Ca^2+^ binding, the AD folded sequentially, templated by the folding of its C-terminal RTX block, with the Ca^2+^-binding segment folding before its N-terminal segment. Selective perturbation of ΔRTX-i/v using a β-hairpin insertion slowed down the folding of ΔRTX-i/v, and subsequently gatekept the folding of AD. Our results indicated that the folding of nonacylated AD is templated by its C-terminal RTX domain. These findings suggested that the templated folding mechanism extends beyond the RTX domain into the adjacent functional AD domain and proceeds hierarchically from the RTX C-terminus to the AD, providing a molecular basis for how CyaA couples secretion to folding to ensure efficient toxin maturation.

The adenylate cyclase toxin (CyaA) secreted by *Bordetella pertussis*, the causative agent of whooping cough, is one of the major virulence factors that facilitate the early bacterial colonization of the human airway (1). CyaA is a representative toxin of the repeats-in-toxin (RTX) leukotoxin family. This large 1706-amino-acid residue-long toxin harbors an N-terminal adenylate cyclase domain and a C-terminal RTX-hemolysin moiety. The C-terminal RTX-hemolysin moiety comprises the pore-forming region, the acylation domain, and the RTX domain (2, 3). The acylation domain (AD) is a conserved functional region found in various bacterial pore-forming RTX toxins, including CyaA and the α-hemolysin (HlyA) of *Escherichia coli* (4, 5). AD is generally associated with toxin activity and membrane-interacting functions (6, 7).

The RTX domain features a characteristic consensus nonapeptide sequence that enables Ca^2+^-driven folding. After being synthesized in the Ca^2+^-depleted bacterial cytosol, RTX remains disordered. Upon secretion into the Ca^2+^-rich extracellular space, RTX folds into a Ca^2+^-loaded β-roll structure. Structural biology and biophysical studies revealed that the folding of the C-terminal assembly of the RTX domain (RTX block v (RTX-v) and the capping structure) scaffold the directional folding of the RTX domain in a Ca^2+^-dependent fashion (8, 9). Single-molecule optical tweezers (OT) studies directly showed that the folding of RTX domains follows a strict series, templated folding mechanism, where the folding starts from the C-terminus of the RTX domain and continues in a series fashion to the N-terminus (10, 11, 12).

The AD, connecting the RTX domain with its N-terminal functional domain, bears 2 conserved acylation sites, K860 and K983, whose palmitoylation is crucial for target cell membrane penetration and toxin activity of CyaA (13). Recently, the three-dimensional structure of the nonacylated AD with the RTX domain was determined using cryo-EM (14). The structure reveals that the AD comprises a non-canonical β-roll structure with 2 extruded β-sheets that bear the 2 acylation sites. The AD connects to the first block of the RTX domain (RTX-i) *via* a linker motif that has high homology to the linker motifs between the RTX blocks.

Despite the three-dimensional structure of AD, the detailed folding mechanism of AD remains poorly understood. Although previous studies implicated that the folding of AD may also follow the templated folding mechanism (10, 15), it remains unknown how the folding signal from the RTX domain is transmitted to the AD to drive AD’s folding. Our previous studies have demonstrated the unique advantages and utility of using OT to study the mechanical unfolding/folding mechanism of the RTX domain (10, 11, 12), including directly monitoring the folding order of individual RTX blocks in real time. Building on these advantages, here we used OT to study the mechanical unfolding and folding mechanisms of the nonacylated AD.

A recent study suggested that the folding of acylated AD may require a properly folded adjacent RTX (15). When coupled with the fact that the folding of the RTX domain follows a series-templated folding mechanism (10), this observation implies that investigating the folding of AD would require a construct harboring the AD and the whole RTX domain. Such an AD-RTX construct will contain ∼1000 amino acid residues. Such a large size makes the biophysical study of AD folding challenging. To alleviate this difficulty, variants in which some RTX blocks are truncated have been engineered and shown to be folded, demonstrating that not all RTX blocks are required for the proper folding of RTX domain (16, 17, 18) and for toxin activity (15, 16). To facilitate the OT study of the folding of nonacylated AD, here we adopted the same truncation strategy. By fusing AD to an engineered truncation RTX variant ΔRTX-i/v, we directly monitored the mechanical unfolding and folding of nonacylated AD using OT. Our results showed that the folding of AD proceeds from the C-terminal to the N-terminal end and that its folding depends on the folding status of its C-terminal adjacent RTX block. These findings indicated that the templated folding mechanism extends beyond the RTX domain to the AD, and the folding of nonacylated AD is conditional upon the correct folding of RTX.

## Results

### Fusion RTX i/v recapitulates the folding behavior of RTX-v

**Figure 1:**
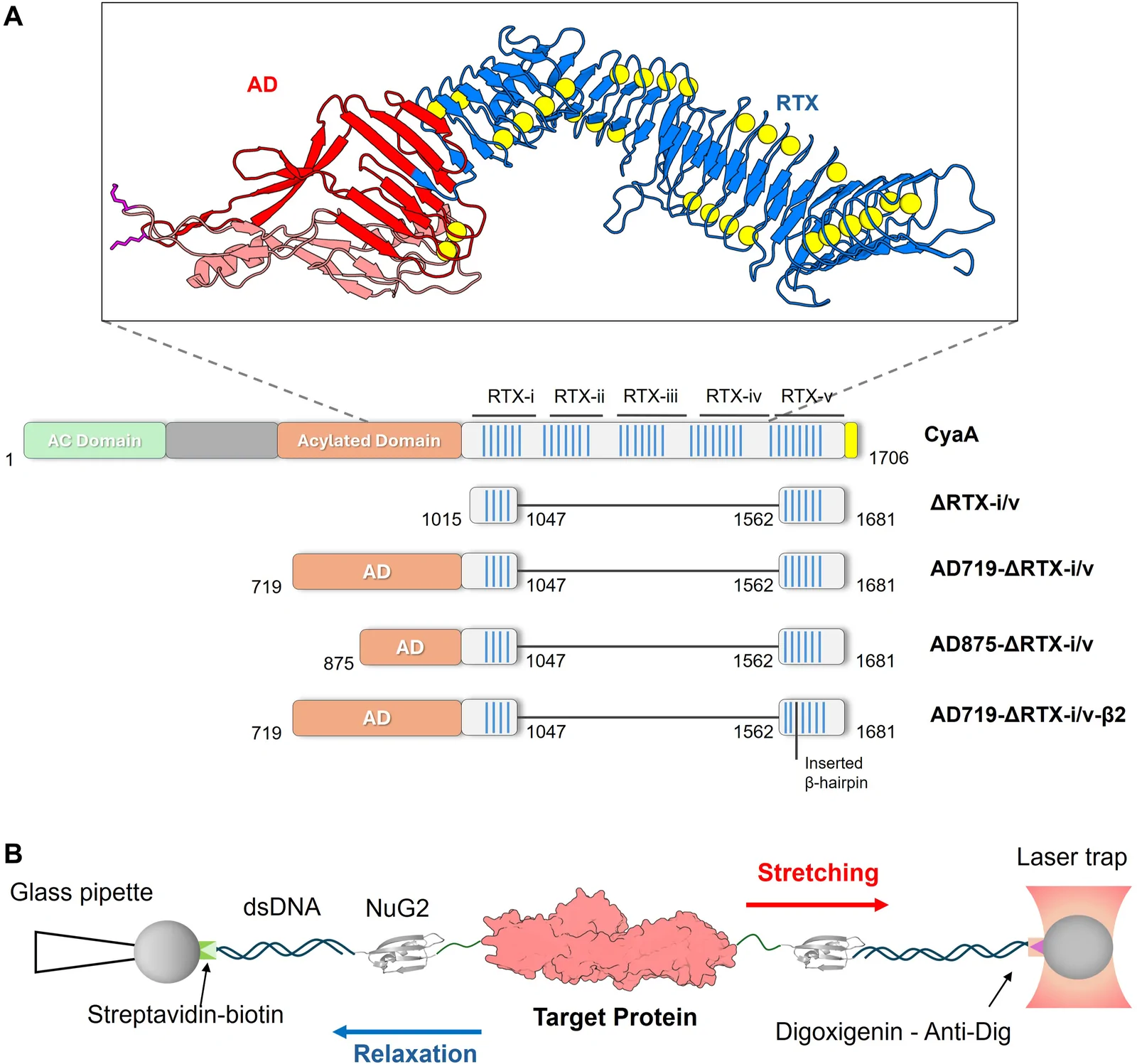
**Schematic of the adenylate cyclase toxin (CyaA), its variants used in this study (A), and the optical tweezers experiment setup (B).** The *blue bars* represent the RTX nonapeptide repeats. The numbers denote the residue index in CyaA. The corresponding name is noted on the *right* side of the construct. The inset is the 3D structure of RTX751 (PDB code: 7USL) containing the acylation domain (colored in *red* and *pink*) and the RTX domain (colored in *blue*). The *yellow* spheres represent the calcium ions.

In previous studies, it was shown that AD undergoes Ca^2+^-driven folding, and its folding can be scaffolded by the C-terminal folded RTX block (15). In addition, truncation of some RTX block in the RTX domain does not necessarily impair the toxin activity of CyaA(16,18). Here we used a previously engineered RTX truncation variant, ΔRTX-i/v, in which residues 1015 to 1047 from RTX-i were fused to residues 1562 to 1681 from RTX-v (15), to engineer the AD-RTX construct for our OT measurements (Fig. 1).

To validate that ΔRTX-i/v folds and to discern its mechanical unfolding/folding signatures, we first carried out OT studies on the standalone ΔRTX-i/v. To this end, we engineered NuG2-ΔRTX-i/v-NuG2, where the well-characterized NuG2 serves as the fingerprint domain for helping identify single-molecule stretching events (19, 20) in OT experiments. The (un)folding of NuG2 displays a contour length increment Δ*Lc* of ∼18 nm, an unfolding force in the range of 30 to 50 pN, and a refolding force of ∼8 pN at the pulling speed of 50 nm/s (21). The observation of 2 NuG2 unfolding events in a given force-distance (F-D) curve ensures that the protein of interest is stretched, and the F-D curve must contain the unfolding/folding signature of the protein of interest.

**Figure 2:**
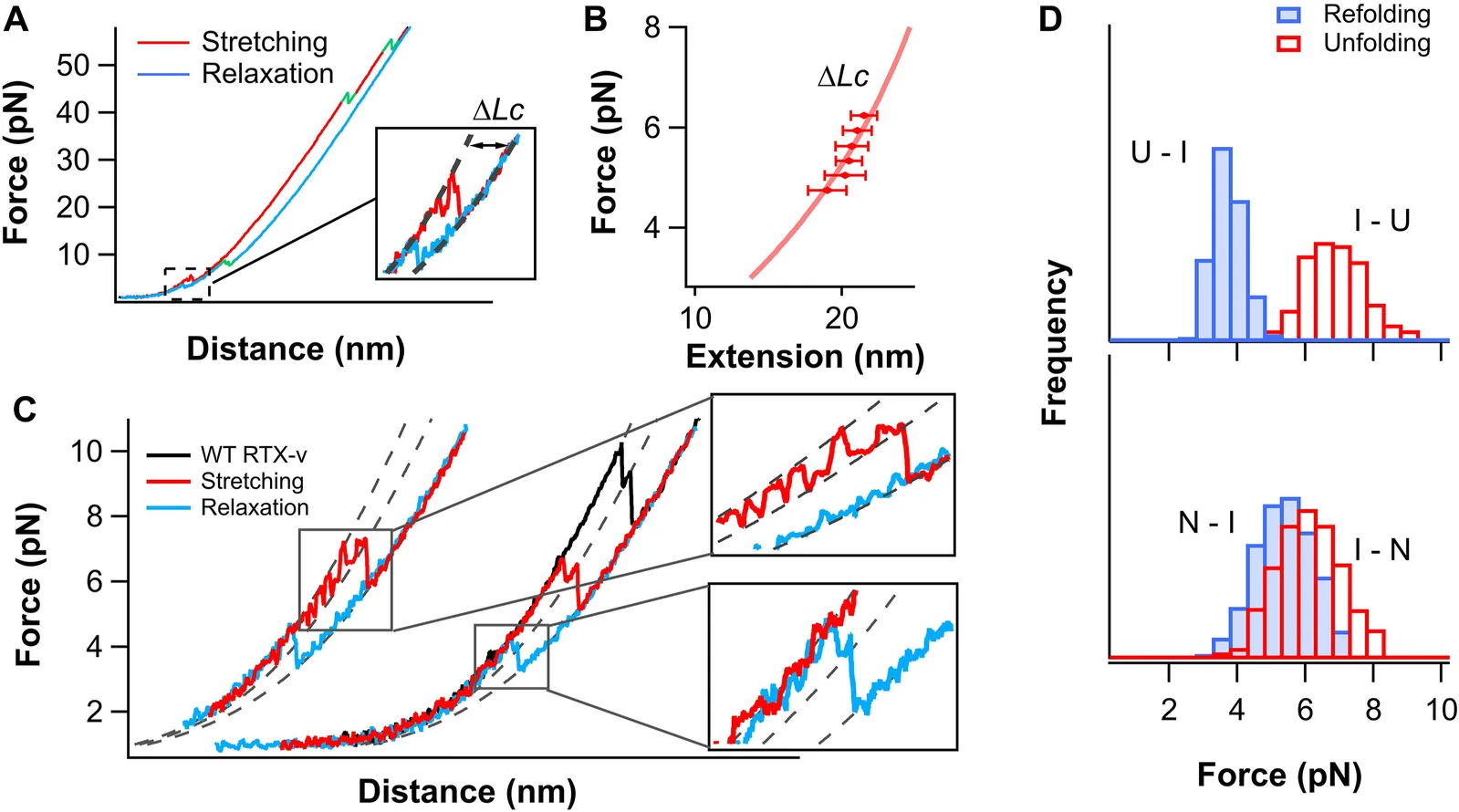
**Mechanical study of ΔRTX-i/v.***A*, representative force-distance curves of NuG2-ΔRTX-i/v-NuG2 at a pulling speed of 50 nm/s, in the presence of 10 mM Ca^2+^. The inset shows the (un)folding events of ΔRTX-i/v at low force region. *B*, force-extension relationship of (un)folding of ΔRTX-i/v. WLC fits (*solid lines*) to the experimental data measured a persistence length of 0.8 nm and a Δ*Lc* of 44.5 nm. *C*, F-D curves of ΔRTX-i/v show three-state unfolding behavior. The black solid curve is a representative unfolding trajectory of WT RTX-v. The *dashed curves* are pseudo-WLC fits. Insets show zoomed-in views of the F-D curves, showing three-state unfolding and folding. *D*, histograms of unfolding and refolding forces of ΔRTX-i/v at a pulling speed of 50 nm/s.

Using the Mini-tweezers setup (http://tweezerslab.unipr.it/cgi-bin/home.pl), we investigated the mechanical (un)folding of ΔRTX-i/v in the presence of 10 mM Ca^2+^, as 10 mM Ca^2+^ saturates the calcium binding of RTX nonapeptides (8). Stretching NuG2-ΔRTX-i/v-NuG2 resulted in 3 unfolding events (Fig. 2*A*, red curve): an unfolding event at ∼6 pN (Fig. 2*A* inset), and 2 unfolding events of the fingerprint domain NuG2 (occurring at >30 pN with a Δ*Lc* of ∼18 nm). Upon relaxation, 3 refolding events were subsequently observed: 2 NuG2 refolding events (with a Δ*Lc* of ∼18 nm and at ∼ 8 pN), and a refolding event at ∼3 pN. The unfolding and folding events at lower forces can be readily attributed to ΔRTX-i/v. Fitting the force-extension relationship of ΔRTX-i/v to the Worm-like chain (WLC) model of polymer elasticity yields a Δ*Lc* of 44.5 ± 0.4 nm (average ± standard deviation) (Fig. 2*B*), which is similar to that of RTX-v (12). Since ΔRTX-i/v has the same number of aa residues as wild type (wt) RTX-v, the observed Δ*Lc* suggested that C-terminal nonapeptide repeats of RTX-v successfully scaffolded the N-terminal RTX repeats from RTX-i in ΔRTX-i/v.

Since the unfolding of ΔRTX-i/v occurred at much lower forces than NuG2, the subsequent stretching–relaxation cycles were limited to below 15 pN so that only ΔRTX-i/v unfolded/refolded, while NuG2 remains folded throughout (Fig. 2*C*). As shown in the F-D curves, ΔRTX-i/v unfolds in 2 steps involving an unfolding intermediate state, with a Δ*Lc1* of 17 nm (native state to the intermediate state, N-I) and Δ*Lc2* of 29 nm (intermediate state to the unfolded state, I-U). This three-state unfolding behavior is similar to that of wt RTX-v (Fig. 2*C*, black curve). It is of note that the N-I transition of ΔRTX-i/v occurred near equilibrium, giving rise to the rapid hopping between the N and I states of ΔRTX-i/v. In contrast, the N-I transition of wt RTX-v occurred at higher forces than ΔRTX-i/v, and did not show hopping, suggesting that RTX-v is more stable than ΔRTX-i/v. Nevertheless, these results indicated that the RTX-v C-terminal nonapeptide sequence successfully scaffolded the folding of the N-terminal nonapeptide repeats from RTX-i, and thus the folded ΔRTX-i/v could template the folding of AD. In addition, the mechanical features of the I-U and U-I transitions in ΔRTX-i/v matched those of wt RTX-v. Our previous study showed that the intermediate state in wt RTX-v corresponds to the C-terminal region of RTX-v (residues 1574–1681, encompassing the C-terminal RTX repeats and the so-called capping structure) (17). Since ΔRTX-i/v contains residues 1574 to 1681 from RTX-v, the folding intermediate state observed in ΔRTX-i/v should correspond to the same structure as the one in RTX-v. It is also evident that ΔRTX-i/v retained the vectorial folding from C-terminus to N-terminus (8, 12).

### The folding of the acylation domain is driven by Ca^2+^ binding

**Figure 3:**
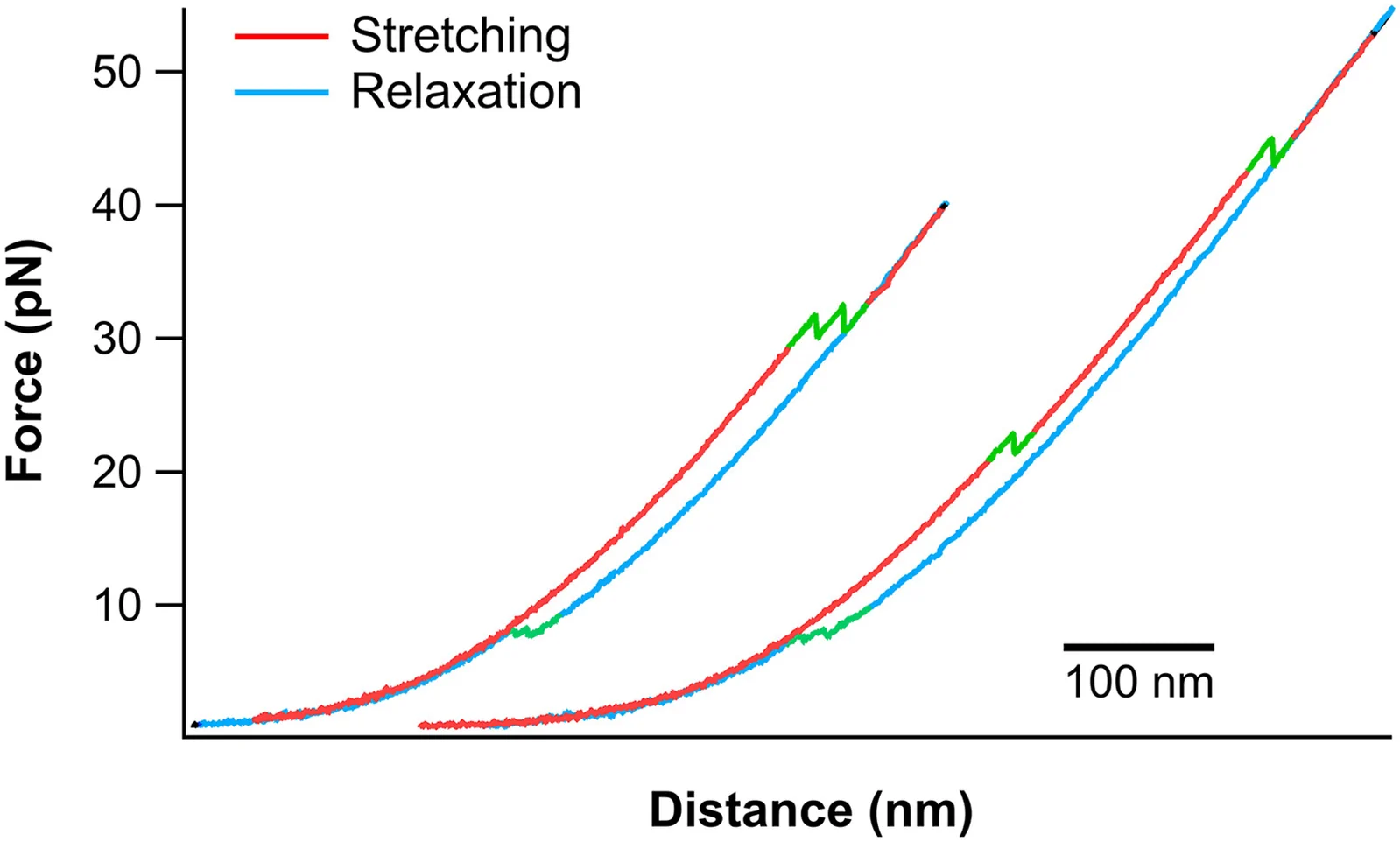
**Representative F-D curves of apo-AD719-ΔRTX-i/v at a pulling speed of 50 nm/s.** The NuG2 events are colored *green*.

Having established the mechanical (un)folding signature of ΔRTX-i/v, we then studied the mechanical unfolding/refolding of nonacylated AD (residues 719–1014) fused to ΔRTX-i/v, denoted as AD719-ΔRTX-i/v, by using the construct NuG2-AD719-ΔRTX-i/v-NuG2. We first stretched NuG2-AD719-ΔRTX-i/v-NuG2 in the absence of Ca^2+^. Figure 3 shows 2 representative F-D curves of stretching-relaxation cycles of the apo-NuG2-AD719-ΔRTX-i/v-NuG2. The F-D curves displayed 2 NuG2 (un)folding events, and no additional unfolding or refolding events were observed, indicating that apo-AD719-ΔRTX-i/v is unfolded and mechanically compliant. A previous study suggested that residues 719 to 770 at the N-terminus of AD may independently adopt a helical motif even in the absence of Ca^2+^ (15). The fact that the F-D data of apo-NuG2-AD719-ΔRTX-i/v-NuG2 (Fig. 3) did not show any unfolding event of AD719-ΔRTX-i/v suggested that even if residues 719 to 770 could fold in the absence of Ca^2+^, its structure is mechanically labile and does not generate any appreciable enthalpic resistance during stretching to give rise to a clear unfolding signature.

**Figure 4:**
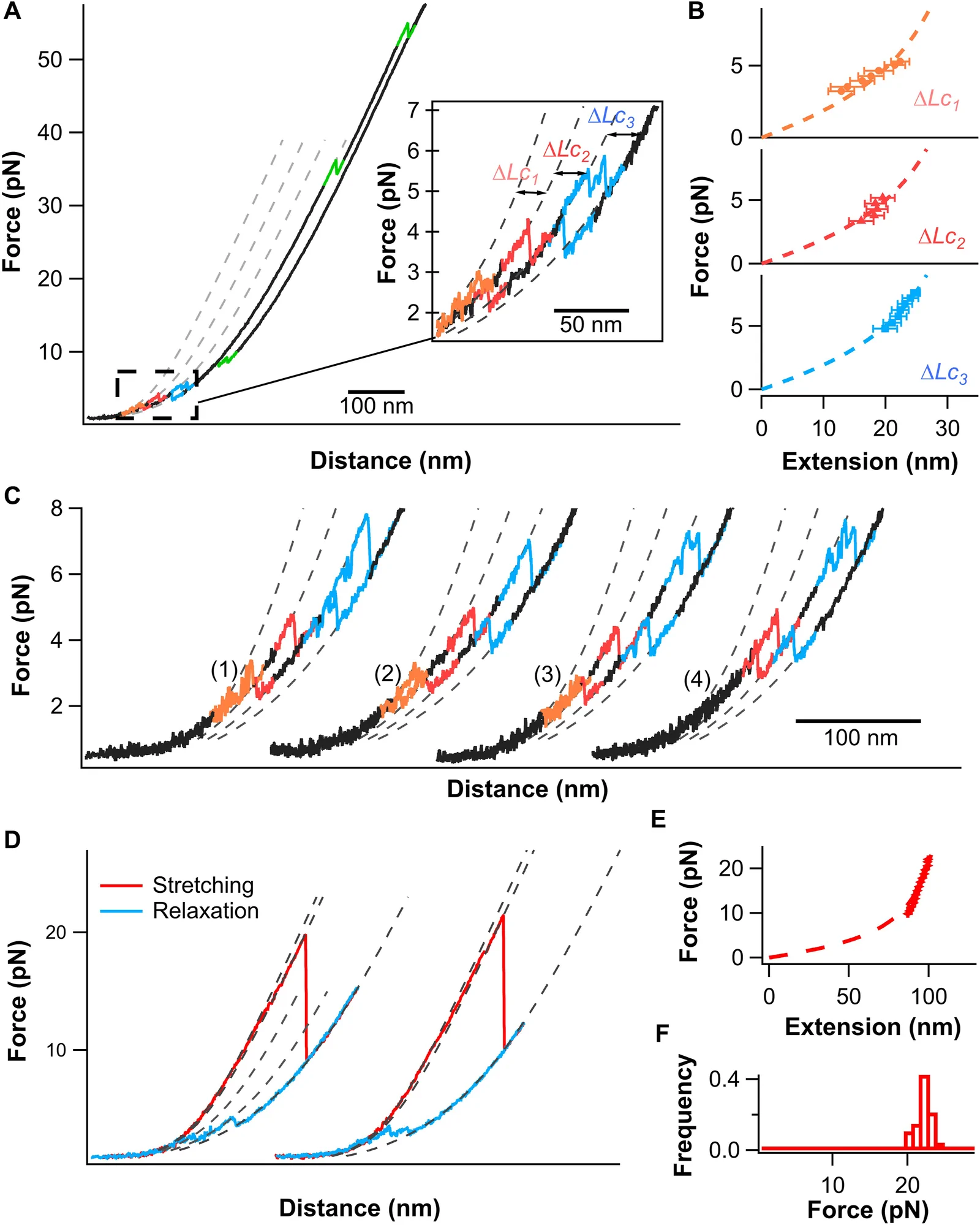
**Mechanical unfolding and refolding of holo-AD719-ΔRTX-i/v.***A*, representative F-D curves of stretching NuG2-AD719-ΔRTX-i/v-NuG2 at a pulling speed of 50 nm/s in the presence of 10 mM Ca^2+^. The NuG2 events are colored *green*. The *grey dashed curves* are pseudo-WLC fittings. The inset shows the 3 (un)folding events that occurred in the low-force regime, highlighted in *orange*, *red*, and *blue*, respectively. *B*, force-extension relationships of (un)folding of AD719-ΔRTX-i/v. WLC fits (*dashed curves*) to the experimental data yield a persistence length of 0.8 nm, Δ*Lc1* of 46.8 ± 1.4 nm, Δ*Lc2* of 45.8 ± 1.2 nm, and Δ*Lc3* of 45.8 ± 0.3 nm. *C*, representative F-D curves of the stretching-relaxation cycle of holo-AD719-ΔRTX-i/v. Cycles one and two show clear 3 (un)folding events, whereas in cycles 3 and 4, only 2 (un)folding events can be clearly identified from the F-D curves. *D*, representative F-D curves show that the holo-AD719-ΔRTX-i/v unfolds in one big step at high force regime. The *grey dashed curves* are pseudo-WLC fittings. *E*, force-extension relationship of one-step unfolding of AD719-ΔRTX-i/v. WLC fits (*dashed curve*) to the experimental data measured a Δ*Lc* of 135.5 ± 0.6 nm. *F*, histogram of the unfolding force of one-step unfolding events observed at a pulling speed of 50 nm/s.

We next examined the mechanical (un)folding of NuG2-AD719-ΔRTX-i/v-NuG2 in the presence of 10 mM Ca^2+^. As shown in Figure 4*A*, the F-D curves of NuG2-AD719-ΔRTX-i/v-NuG2 in the presence of 10 mM Ca^2+^ showed 3 (un)folding events in addition to the 2 NuG2 events, which can be readily assigned to the unfolding/refolding of AD719-ΔRTX-i/v. Since the total Δ*Lc* of the 3 (un)folding events (∼138 nm) is significantly larger than that of ΔRTX-i/v, these 3 (un)folding events must involve the (un)folding of AD719. Since apo-AD719-ΔRTX-i/v is unfolded while the holo form is folded, this result indicates that the folding of the AD is also driven by Ca^2+^-binding, similar to the RTX domain.

The (un)folding of AD719-ΔRTX-i/v showed 3 major events (Fig. 4*A* inset and Fig. 4*C*). Fitting the force-extension relationships to the WLC model yielded Δ*Lc1* of 46.8 ± 1.4 nm, Δ*Lc2 of* 45.8 ± 1.2 nm, and Δ*Lc3 of* 45.8 ± 0.3 nm, respectively (Fig. 4*B*). Notably, although the 3 unfolding events showed similar Δ*Lc,* they displayed distinct mechanical signatures. The first unfolding event, colored in orange, occurs at around 3 pN, while the second unfolding event, colored in red, occurs at ∼4 pN and shows a clear “rip”. And the third unfolding event, colored in blue, occurs at ∼6 pN and displays a characteristic unfolding intermediate state, and these signatures are similar to those of ΔRTX-i/v (Fig. 2). Thus, the third unfolding event can be readily assigned to ΔRTX-i/v, and the first and second unfolding events can thereby be assigned to AD. The refolding of AD719-ΔRTX-i/v proceeds in the reverse order of the unfolding: AD unfolds first, and the unfolding of ΔRTX-i/v follows. These results suggest that AD unfolds prior to ΔRTX-i/v, and AD refolds after ΔRTX-i/v has folded.

It is of note that in approximately 51% of trajectories (292 out of 576), the first unfolding event could not be clearly resolved in the F-D curves (Fig. 4*C*, cycles 3 and 4). Since the first unfolding event that can be resolved occurred at low forces ∼3 pN, it is possible that some events occurred at forces lower than 3 pN. Such a low force approached the detection limit of our OT setup, making it not possible to clearly resolve these unfolding events.

In addition, in approximately 8% of trajectories (48 out of 576), the unfolding of AD719-ΔRTX-i/v occurred at considerably higher force (with an average of 21 pN) and in one step (Fig. 4, *D* and *F*). Fitting the force-extension relationship to the WLC model yielded a Δ*Lc* of ∼135.5 ± 0.6 nm (Fig. 4*E*). Notably, prior to this major unfolding event, a gradual extension of ∼ 10 nm is observed, leading to a total Δ*Lc* of ∼ 145 nm. The AD719-ΔRTX-i/v is 449 aa residues long. Our previous OT study on RTX-v showed that residues 1665 to 1681 in RTX-v unfold at low forces (below our OT detection limit) and thus do not contribute to ΔLc of RTX-v(12). Therefore, unfolding AD719-ΔRTX-i/v should result in a Δ*Lc* of 146.3 nm ((449–17)∗ 0.36 nm/aa – 9.2 nm = 146.3 nm, where 9.2 nm is the distance between residues 719 and 1664), in good agreement with the experimentally measured Δ*Lc*.

To examine whether the one-step unfolding pathway was dependent on the folding history, we examined the refolding traces preceding these one-step unfolding events. The preceding relaxation trajectories showed 3 sequential refolding events, and no obvious difference with other observed refolding traces was observed, suggesting that the one-step unfolding state is accessible from the fully folded conformation. Moreover, these one-step unfolding events were observed in different tethers and occurred stochastically during consecutive stretching-relaxation cycles in the same molecule, indicating that these events may arise from a subpopulation that is of considerably higher mechanical stability, and this subpopulation shuffles with the major population that is less stable. It is of note that the one-step unfolding events were not observed in the relaxation curves, namely, no single step refolding event was detected upon relaxation following one-step unfolding. Instead, the subsequent relaxation traces showed the same sequential three-step refolding pattern observed after three-step unfolding.

### The Ca^2+^-binding segment of AD folds independently when its C-terminal RTX block is folded

The OT results on AD719-ΔRTX-i/v showed that AD unfolds prior to the ΔRTX-i/v domain and refolds after the ΔRTX-i/v domain, suggesting that the folding of AD is dependent upon the folding of ΔRTX-i/v. However, the folding pathway of AD itself is yet to be elucidated, and it remains unknown if the series templated folding mechanism found for the RTX domain (10) also extends to AD.

Based on the cryo-EM structure (14) and the amino acid sequence, AD can be divided into 2 main parts: the N-terminal segment (residues 719–874) and the Ca^2+^-binding segment (CaBS) (residues 875–1014). CaBS contains 3 non-canonical RTX-like Ca^2+^-binding motifs binding two Ca^2+^ ions, and the linker motif between CaBS and ΔRTX-i/v also shares a high sequence and structural homology with the linker motifs connecting RTX blocks (14, 22). Therefore, it is reasonable to speculate that the templating mechanism found in the RTX domain also applies to the folding of AD. If this hypothesis holds, the folding pathway of AD should also follow a strict folding hierarchy, in which the C-terminus folds first, followed by the folding of the N-terminus. To test this hypothesis, we constructed AD875-ΔRTX-i/v, which comprises CaBS (residues 875–1014) of AD and ΔRTX-i/v.

**Figure 5:**
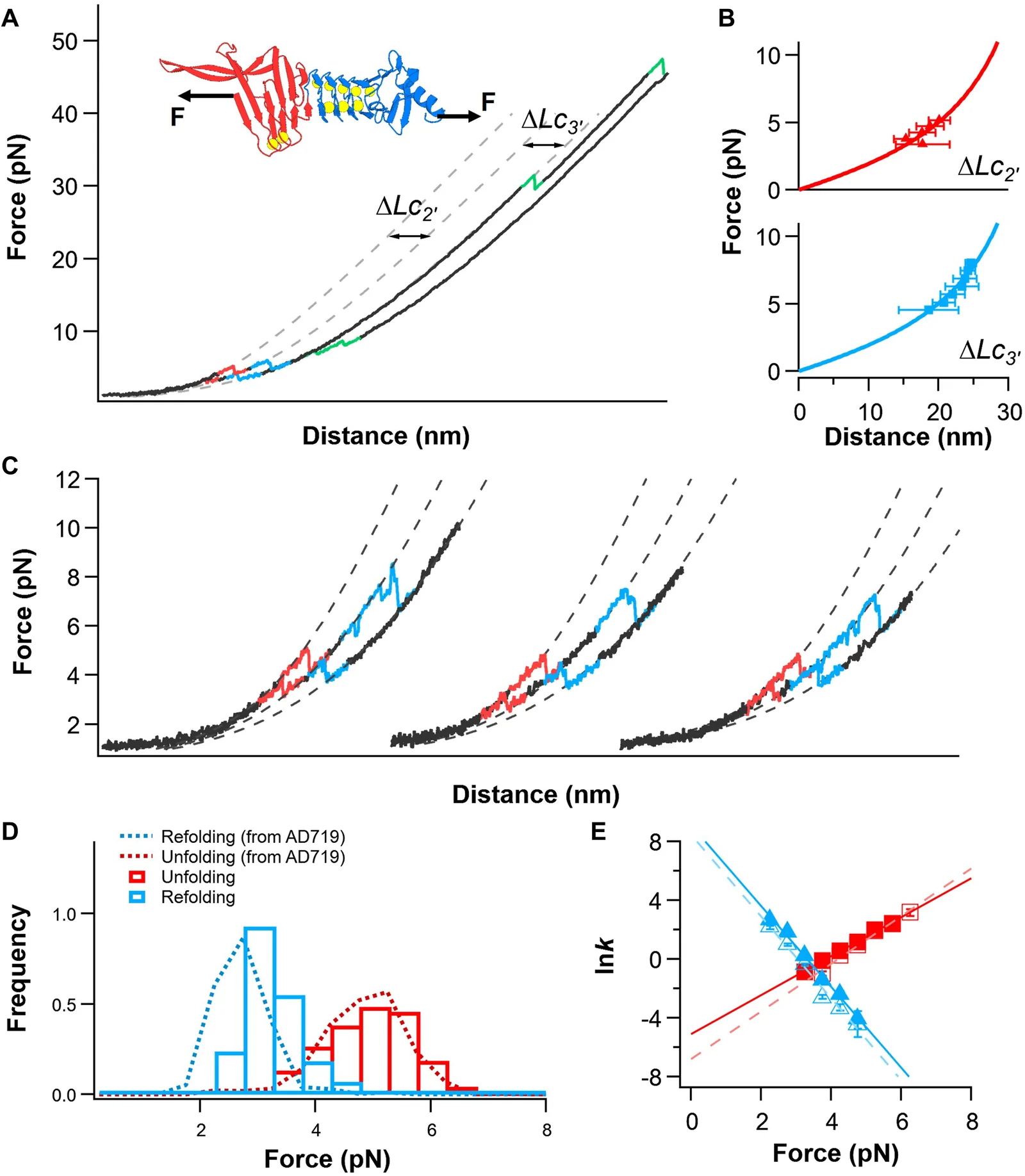
**Mechanical study of AD875-ΔRTX-i/v with the presence of 10 mM Ca^2+^.***A*, representative F-D curves of NuG2-AD875-ΔRTX-i/v-NuG2 at a pulling speed of 50 nm/s. The NuG2 events are colored *green*. The *grey dashed curves* are pseudo-WLC fittings. *B*, Force-extension relationships of (un)folding of AD875-ΔRTX-i/v. WLC fits (*dashed curves*) to the experimental data yield a persistence length of 0.8 nm, Δ*Lc1′* of 45.7 ± 1.4 nm, and Δ*Lc2′* of 45.6 ± 0.4 nm. The 3D structure of AD875-ΔRTX-i/v was predicted by ColabFold (31). *C*, Representative stretching-relaxation cycles show the (un)folding of AD875-ΔRTX-i/v. *D*, Histograms of unfolding and refolding forces of the CaBS at a pulling speed of 50 nm/s. The bars represent the unfolding (n = 514) and refolding force (n = 486) distribution of the CaBS from the AD875-ΔRTX-i/v. The *dashed curves* represent unfolding (n = 621) and refolding force (n = 627) distribution of the CaBS from the AD719-ΔRTX-i/v. *E*, the unfolding/refolding rates measured at different forces and fits to the Bell-Evans model. The solid squares and triangles represent the rates measured for the CaBS from the AD875-ΔRTX-i/v, whereas the open squares and triangles represent the rates measured for the CaBS from the AD719-ΔRTX-i/v.

Stretching AD875-ΔRTX-i/v yields 2 (un)folding events (Fig. 5*A*). The first unfolding event (colored in red) occurred at ∼5 pN in an all-or-none fashion, and the second one (colored in blue) occurred ∼7 pN and involved a clear unfolding intermediate. WLC fits to the force-extension curves measured Δ*Lc* of 45.7 ± 1.4 nm and 45.6 ± 0.4 nm for the 2 unfolding events, respectively (Fig. 5*B*). The blue unfolding event displayed signatures matching those of ΔRTX-i/v and thus can be assigned to ΔRTX-i/v. The red unfolding event can be readily assigned to CaBS.

Comparing the F-D curves of AD875-ΔRTX-i/v with those of AD719-ΔRTX-i/v, it is evident that the unfolding event of CaBS and ΔRTX-i/v in AD875-ΔRTX-i/v matches the second and third unfolding events of AD719-ΔRTX-i/v, and the first unfolding event in AD719-ΔRTX-i/v is missing in AD875-ΔRTX-i/v. Therefore, the orange unfolding event in AD719-ΔRTX-i/v corresponds to the unfolding of the N-terminal segment of AD. These results clearly indicate that the CaBS folds first, followed by the N-terminal segment, while the N-terminal segment unfolds first, followed by CaBS. This sequence showed a clear sequential/vectorial folding/unfolding pathway of AD719. Moreover, the average unfolding force for the CaBS in AD875-ΔRTX-i/v is 4.7 pN, and the average refolding force is 2.9 pN. These values are in close agreement with the forces measured in AD719-ΔRTX-i/v, with unfolding and refolding forces of 4.9 pN and 2.7 pN, respectively, indicating that the folding of the CaBS does not depend on the N-terminal segment of AD (Fig. 5*D*).

We further characterized the folding kinetics of the CaBS in AD875-ΔRTX-i/v. Using the Oesterhelt method (23), the (un)folding kinetics was extracted from the F-D curves. Fitting to the Bell-Evans model (24, 25, 26) to the force dependency of the (un)folding rate yielded an unfolding rate constant and refolding rate constant at zero force of 0.6 × 10^-3^ s^-1^ and 8.9 × 10^3^ s^-1^, respectively. These values are similar to those measured in AD719-ΔRTX-i/v (Fig. 5*E*), suggesting that truncation of the N-terminal segment does not affect the folding kinetics or mechanical stability of CaBS of AD. Together, these results showed that the folding of CaBS is scaffolded by its C-terminal RTX block, and suggested that the folding of AD follows a hierarchical folding mechanism in which AD folding proceeds from the C-terminus to the N-terminus.

### The templated folding mechanism extends from the RTX domain to the AD

From the F-D curves, it is clear that the AD719-ΔRTX-i/v follows a sequential (un)folding hierarchy: the AD unfolds first from its N-terminal segment, then CaBS, and finally the RTX domain, whereas refolding proceeds in the reverse order. This observation is consistent with the templated folding mechanism (10). To further validate this templated folding mechanism, we engineered a loop insertion variant of ΔRTX-i/v to directly demonstrate that the folding of AD719 is templated by the complete folding of ΔRTX-i/v. To this end, we inserted a β-hairpin between residues S1573 and L1572 of ΔRTX-i/v to selectively slow down the folding of the N-terminal RTX repeats in ΔRTX-i/v. This insertion variant was designed to selectively slow the folding of the N-terminal RTX repeats while minimizing perturbations to the overall structure of AD719-ΔRTX-i/v. The second β-hairpin from the small protein GB1, which is 15 residues long and flanked by an AA dipeptide linker at both the N- and C-termini, was chosen to be inserted. This variant is termed AD719-ΔRTX-i/v-β2. This β hairpin is marginally stable in isolation and adopts ∼40% of a native-like β-hairpin structure at room temperature (27, 28), thus it is unlikely that its insertion would disrupt the original folded structure of ΔRTX-i/v (29). Previous studies on RTX-v showed that the insertion site can tolerate sequence insertion without altering the β-roll structure of the folded RTX-v block (11). Since the hairpin is inserted between residues 1572 and 1573, right at the N-terminal end of the RTX-v folding intermediate (residues 1574–1681), the loop insertion should not affect the folding of the folding intermediate state. Instead, it could slow down the folding of the N-terminal part of ΔRTX-i/v due to the increased entropic penalty associated with closing the inserted hairpin. It would be expected that the slower refolding of the N-terminal part of ΔRTX-i/v would slow down the folding of AD if the templated folding mechanism governed the folding of AD-RTX.

**Figure 6:**
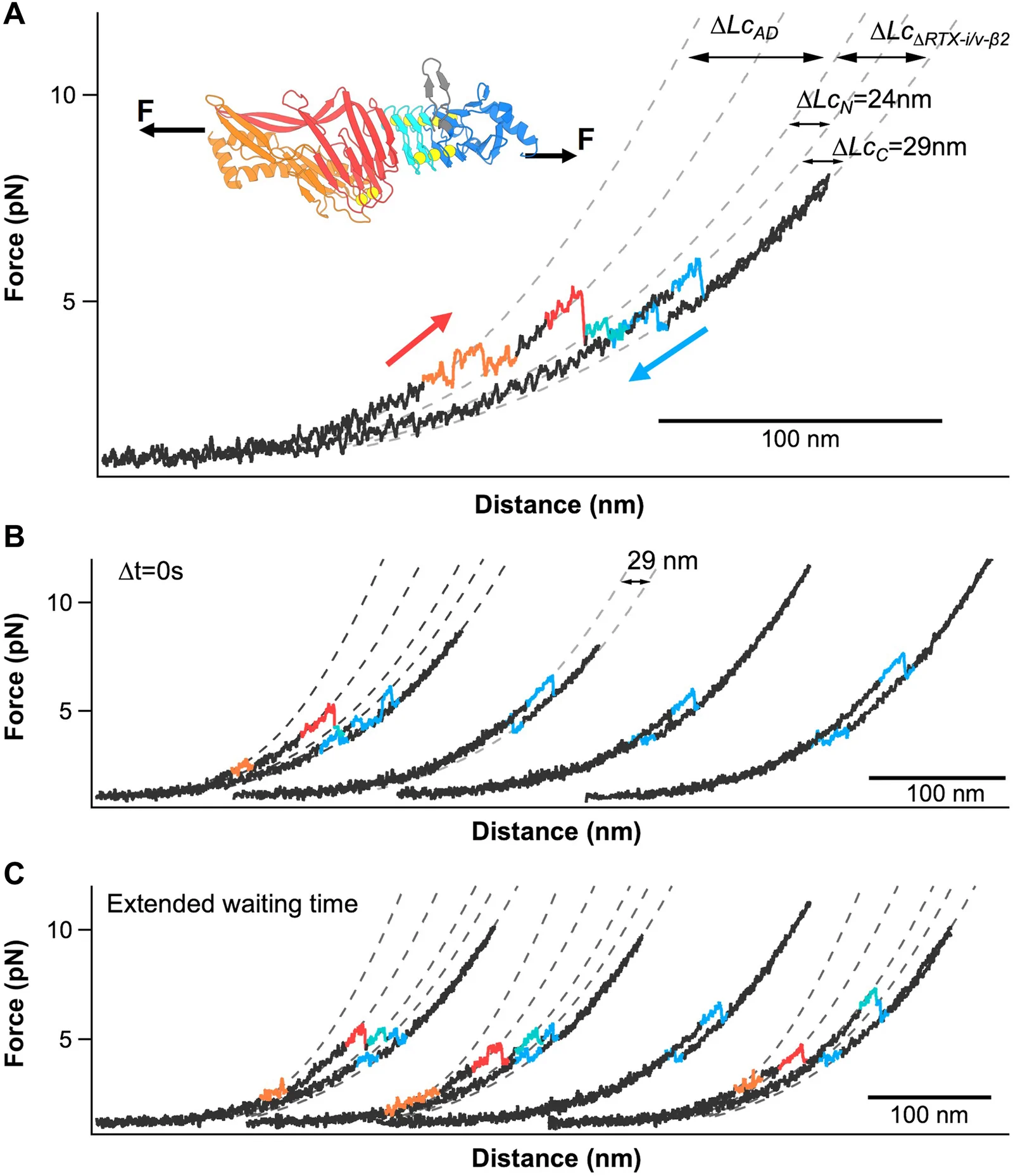
**Mechanical study of AD719-ΔRTX-i/v-β2 in the presence of 10 mM Ca^2+^.***A*, representative F-D curves of AD719-ΔRTX-i/v-β2 at a pulling speed of 50 nm/s. The *grey dashed curves* are pseudo-WLC fittings. The inset is the ColabFold predicted structure of AD719-ΔRTX-i/v-β2. The inserted GB1 segment between L1572 and S1573 is colored in *grey*. The *red* and *blue arrows* indicate the stretching and relaxation direction, respectively. The unfolding events of the N-terminal segment and CaBS of AD are colored in *orange* and *red*, respectively. The unfolding event of ΔRTX-i/v-β2 from its native state to the intermediate state is colored in *cyan*, and the (un)folding events of the intermediate state of ΔRTX-i/v-β2 are colored *blue*. *B* and *C*, F-D curves of consecutive stretching-relaxation cycles with no waiting time (Δt) at zero force (*B*), and with extended waiting time (*C*).

Figure 6*A* shows the F-D curves of stretching AD719-ΔRTX-i/v-β2. The first 2 unfolding events exhibited Δ*Lc* of 47 nm and 46 nm, corresponding to the unfolding of the N-terminal segment and CaBS of AD, respectively. The remaining 2 events correspond to the unfolding of ΔRTX-i/v-β2. The blue event at ∼7 pN with a Δ*Lc* of 29 nm corresponds to the unfolding of the intermediate state of RTX-v (residues 1574–1681), the cyan event at ∼4 pN corresponds to the unfolding of ΔRTX-i/v-β2 from its native state to the intermediate state. It is of note that the N-I transition for ΔRTX-i/v-β2 occurred at a lower force than that of wt RTX-v but with a longer Δ*Lc* (Δ*Lc* of 24 nm for ΔRTX-i/v-β2 vs 17 nm for wt RTX-v). The increment of Δ*Lc* (7 nm) matched well with the length of the inserted β hairpin (19 aa ∗ 0.36 nm/aa=6.8 nm). The decreased unfolding force of ΔRTX-i/v-β2 is likely due to the destabilization caused by the loop insertion.

In subsequent stretching–relaxation cycles, we observed direct supporting evidence for the templated folding mechanism for AD-ΔRTX-i/v. When the fully unfolded AD-ΔRTX-i/v-β2 was relaxed to zero force and stretched again without any waiting time at zero force (Δt = 0s), only the unfolding and refolding events of the folding intermediate state of RTX-v were observed (Fig. 6*B*), suggesting that AD and the N-terminal repeats of ΔRTX-i/v did not fold. This result indicated that the folding intermediate state of RTX-v alone is not sufficient to template the folding of AD. When the unfolded molecule was allowed to relax at zero force for 30 s (Δt = 30s), a significant percentage of trajectories displayed the unfolding events of AD and the whole ΔRTX-i/v, however, only the refolding of the intermediate state was observed on the relaxation traces (Fig. 6*C*). This result indicates that the loop insertion significantly slowed down the folding of the N-terminal nonapeptide repeats of ΔRTX-i/v. Notably, the unfolding events of AD appeared always together with the fully folded ΔRTX-i/v-β2, and we never observed AD unfolding event in a F-D curve with only the unfolding event of the folding intermediate of ΔRTX-i/v-β2. Clearly, the folding of AD is conditional upon the folding of the whole ΔRTX-i/v-β2, and as soon as ΔRTX-i/v-β2 had refolded, AD could refold quickly. These results clearly indicated that the folding of AD is templated by the folding of its C-terminal RTX block. Considering the domain structure of CyaA, it becomes evident that the series templated folding mechanism extends from the entire RTX domain to the AD domain.

## Discussion

Elucidating the folding pathway of the AD is essential for understanding how the toxin maturation is coordinated with its secretion. A previous single-molecule study demonstrated that the RTX domain of CyaA folds *via* a vectorial C-to-N-terminal folding pathway (8, 10). The results presented here suggest that the nonacylated AD follows the same C-to-N-terminal folding pathway, extending this mechanism beyond the RTX domain itself.

Based on the folding behavior of AD719-ΔRTX-i/v and AD875-ΔRTX-i/v, we propose a hierarchical (un)folding pathway for the nonacylated AD. Upon scaffolding by its C-terminal RTX block, the Ca^2+^-binding segment of the AD folds first, followed by the N-terminal segment of AD. Conversely, the unfolding begins with the N-terminal segment and is followed by the Ca^2+^-binding segment. Notably, the Ca^2+^-binding segment of the AD lacks the canonical RTX nonapeptide Ca^2+^-binding motif, yet exhibits Ca^2+^-driven folding behavior, suggesting that it may serve as an intermediate that nucleates the folding of the N-terminal segment of AD.

These findings align with a recent study showing that the Ca^2+^-loaded AD acts as a regulatory calcium lock for membrane penetration of RTX cytolysins. In that study, disruption of the Ca^2+^-coordinating N936 residue was proposed to block propagation of the Ca^2+^-driven C-to-N folding signal beyond residue 936, thereby impairing formation of the Ca^2+^-binding sites in CaBS and folding of the N-terminal portion of the acylation domain (22). Our single-molecule results provide direct support for this model by showing that the CaBS folds first and is required for the templated folding of the N-terminal segment of the acylation domain.

A recent SAXS study revealed that the nonacylated AD-RTX alternates its conformation between a compact conformation and a more extended one (15). We hypothesize that this conformational heterogeneity may bear relevance to the OT results observed in this study. Specifically, we speculate that when the N-terminal segment adopts a more extended conformation, it may be mechanically too compliant to generate a detectable unfolding event, which could account for the ∼51% of trajectories in which its unfolding was not resolved (Fig. 4). Conversely, the mechanically stable conformation observed in our OT study (∼8% of unfolding trajectories with one-step unfolding at high force) might correspond to the compacted AD conformation. Nevertheless, further characterization is required to test this hypothesis.

Moreover, it is important to emphasize that the current study is focused on the nonacylated AD. Although previous studies indicated that post-translational acylation of AD is essential for the folding and stability of AD(15), our OT results showed that the nonacylated AD can fold, raising the question how post-translational acylation at K860 and K983 modulates the folding of the AD. Acylation occurs inside the bacterial cytosol before secretion, and was shown to be critical for toxin activity (15, 30). It will be important to examine if and how the acylation will affect the templated folding mechanism in the acylated AD. In addition, while the truncated RTX scaffold ΔRTX-i/v, recapitulated the folding hierarchy of the native RTX block, it lacks RTX block ii-iv. It remains to be examined if these blocks contribute to AD folding efficiency in the full-length toxin, as the stabilization by the neighboring RTX block is significant in the RTX domain.

## Conclusion

Using OT-based single-molecule technique, we directly investigated the folding mechanism of the nonacylated AD domain of CyaA using a truncated RTX scaffold. Our results revealed that the AD folds through a hierarchical pathway that proceeds from the C-terminus toward the N-terminus and is templated by its C-terminal adjacent RTX scaffold. The Ca^2+^-binding segment (residues 875–1014) of the AD folds first upon the folding of the RTX-scaffold, followed by the folding of the mechanically more compliant N-terminal segment (residues 719–874). This hierarchical folding pathway suggests that the templated folding mechanism previously identified for the RTX domain extends into the acylation domain and may coordinate toxin maturation. These findings provide new mechanistic insights into how vectorial folding may cooperate to ensure efficient assembly and activation of the adenylate cyclase toxin and may offer new insight into designing therapeutic strategies to combat pertussis.

## Experimental procedures

### Protein engineering and purification

The gene encoding the fusion ΔRTX-i/v was amplified from pT7CT7/AD-ΔRTX-i-v reported in Ref 15. using polymerase chain reaction (PCR). The gene encoding AD719-ΔRTX-i/v and AD719-ΔRTX-i/v-β2 were constructed *via* the Gibson assembly method. The gene encoding AD875-ΔRTX-i/v was amplified from the AD719-ΔRTX-i/v gene. The *Bam*HI (5′) and *Kpn*I (3′) restriction sites were incorporated into the genes using custom-designed primers for the PCR reaction. The amplified genes encoding the ΔRTX-i/v, AD719-ΔRTX-i/v, AD875-ΔRTX-i/v, and AD719-ΔRTX-i/v-β2 were subsequently ligated into our custom-engineered vector, pETcc, which harbors a cassette encoding 5′ Cys-NuG2 and 3′ NuG2-Cys, *via Bam*HI and *Kpn*I restriction sites. The obtained genes are pETcc/Cys-NuG2-ΔRTX-i/v-NuG2-Cys, pETcc/Cys-NuG2-AD719-ΔRTX-i/v-NuG2-Cys, pETcc/Cys-NuG2-AD875-ΔRTX-i/v-NuG2-Cys, and pETcc/Cys-NuG2-AD719-ΔRTX-i/v-β2-NuG2-Cys.

Recombinant proteins were overexpressed in the *E. coli* strain BL21(DE3) grown at 37 °C, 225 r.p.m. in 200 ml of 2.5% LB medium containing 100 mg/L ampicillin. Expression was induced with 1 mM IPTG (ThermoFisher Scientific) when the optical density (OD600) of the culture reached 0.6. Overexpression continued for 4 h at 37 °C, 225 r.p.m. *E. coli* cell pellets were collected by centrifugation at 5000 rpm at 4 °C for 10 min.

For Cys-NuG2-ΔRTX-i/v-NuG2-Cys, protein extraction was performed using the lysozyme lysis method, followed by purification *via* Co^2+^ affinity chromatography. The protein was eluted and stored in elution buffer (10 mM PBS, 300 mM NaCl, 250 mM imidazole). The purified apo-form RTX protein solution was diluted to approximately 1.0 mg/ml and stored at −80 °C.

For protein constructs involving AD, including Cys-NuG2-AD719-ΔRTX-i/v-NuG2-Cys, Cys-NuG2-AD875-ΔRTX-i/v-NuG2-Cys, and Cys-NuG2-AD719-ΔRTX-i/v-β2-NuG2-Cys, the corresponding *E. coli* cell pellet was first lysed with lysozyme, and the lysate was centrifuged at 12,000 r.p.m for 30 min at 4 °C. The pelleted inclusion bodies were resuspended in TNU buffer (50 mM Tris-HCl, 150 mM NaCl, 8M Urea, pH=8.0). The urea extracts were clarified by centrifugation at 25,000×*g* for 30 min at 4 °C. The clarified urea extracts were loaded on a Co^2+^-NTA column pre-equilibrated with the TNU buffer. The purified proteins were eluted from the column with the TNU-elution buffer (TNU buffer supplemented with 250 mM imidazole). The urea-denatured proteins were then refolded on the Superdex 200 Increase 10/300 GL size-exclusion column (Cytiva), equilibrated with PBS buffer. Collected fractions containing the target protein were confirmed by SDS-PAGE and stored at −80 °C.

### Preparation of DNA-protein

DNA handles were prepared as previously described. Briefly, biotin- and digoxigenin-modified DNA handles were generated by standard polymerase chain reaction (PCR) by using the pGEMEX-1 plasmid as the template, and a 5′ biotin- or a 5′ digoxigenin-modified forward primer and a 5′ NH2-modified reverse primer (purchased from Integrated DNA Technologies Inc., San Jose, CA). After PCR amplification, the DNA handles were purified using the QIAquick PCR purification kit (QIAGEN) and then reacted overnight with 4-(N-maleimidomethyl) cyclohexane-1-carboxylic acid N-hydroxysuccinimide ester (SMCC; Sigma-Aldrich) to introduce a terminal maleimide group. 1 μl 1.5 μM maleimide-functionalized DNA handles were conjugated to 1 μl 5 μM freshly expressed proteins *via* thiol–maleimide chemistry by overnight incubation. The resulting DNA–protein chimeras were diluted to ∼10 nM and stored at −80 °C before use.

### Single-molecule optical tweezers experiment

The optical tweezers experiments were carried out on an Minitweezers setup. 1 μl streptavidin-coated polystyrene beads (1% w/v 1 μm, Spherotech Inc) was diluted by 3 ml Tris–HCl buffer (20 mM Tris, 150 mM NaCl, pH 7.4) and injected into the fluid chamber. One single streptavidin-coated bead was trapped by the laser beam and moved onto the tip of a micropipette. The bead was then immobilized on the pipette by applying a vacuum. 4 μl of anti-digoxigenin–coated polystyrene beads (0.1% w/v, 2 μm, Spherotech Inc) was mixed with 1 μl ∼10 nM DNA-protein chimera and incubated for 30 min. The target-protein-coated anti-dig beads were diluted by 3 ml Tris–HCl buffer (20 mM Tris, 150 mM NaCl, pH 7.4) and injected into the fluid chamber. The sample-coated anti-dig bead was captured by the optical trap and brought into contact with the streptavidin bead held by the micropipette, thereby establishing the bead-DNA–protein dumbbell through the biotin–streptavidin interaction. By manipulating the laser trap, the target protein could be stretched or relaxed to induce mechanical unfolding or folding. For experiments on holo-AD-RTX variants, Tris–HCl buffer supplemented with calcium (20 mM Tris, 150 mM NaCl, 10 mM CaCl_2_, pH 7.4) was used.

## Data availability

All data pertinent to this work are contained within this manuscript or available upon request. For requests, please contact: Hongbin Li, hongbin@chem.ubc.ca.

## Conflict of interest

The authors declare that they have no conflicts of interest with the contents of this article.
